# A high platelet-to-lymphocyte ratio predicts all-cause mortality and cardiovascular mortality in maintenance hemodialysis patients

**DOI:** 10.1080/0886022X.2023.2258228

**Published:** 2023-09-19

**Authors:** Yanping Zhang, Aihong Zhang, Lin Wei, Kaiming Ren, Qian Wang, Bing Shao, Chen Zhao, Zhuo Ren, Jiuxu Bai, Ning Cao

**Affiliations:** Department of Blood Purification, General Hospital of Northern Theatre Command, Shenyang, Liaoning, China

**Keywords:** Platelet-to-lymphocyte ratio, maintenance hemodialysis, all-cause mortality, cardiovascular mortality, neutrophil-to-lymphocyte ratio

## Abstract

**Purpose:**

The aim of this study was to further assess whether the platelet-to-lymphocyte ratio (PLR) is independently associated with all-cause mortality and cardiovascular mortality in maintenance hemodialysis (MHD) patients.

**Methods:**

From January 1, 2014, to December 31, 2014, patients undergoing regular hemodialysis in the Blood Purification Center of the General Hospital of Northern Theatre Command were retrospectively selected. A total of 303 MHD patients were enrolled in accordance with the inclusion and exclusion criteria. For each patient, the endpoint of follow-up was either death or December 31, 2021. The primary endpoints were all-cause and cardiovascular death. A receiver operating characteristic (ROC) curve was drawn to detect the predictive ability of PLR, and the optimal critical value of PLR was determined to be 107.57. Kaplan–Meier curves and Cox proportional analysis were used to assess the prognostic value of PLR. We used the same method to evaluate the correlation between the neutrophil-to-lymphocyte ratio (NLR) and the prognosis of MHD patients.

**Results:**

At the end of follow-up, 128 MHD patients had progressed to all-cause death, and 73 MHD patients had progressed to cardiovascular death. In multivariate Cox regression, both the high PLR group and the high NLR group were independently associated with all-cause mortality (HR 2.608, 95% CI 1.579–4.306, *p* < .001 vs. HR 1.634, 95% CI 1.023–2.610, *p* = .04). Only high PLR expression was associated with cardiovascular mortality (HR 3.379, 95% CI 1.646–6.936, *p* = .001).

**Conclusions:**

High PLR levels can independently predict all-cause and cardiovascular mortality in MHD patients.

## 1. Introduction

With the continuous progress of blood purification therapy, the survival status of maintenance hemodialysis (MHD) patients has improved, but the mortality rate is still much higher than that of the normal population. Cardiovascular disease (CVD) is considered a common complication and the main cause of death in MHD patients [1]. However, some conventional cardiovascular risk factors are poor predictors of mortality in patients on MHD [2]. A growing number of studies have identified nontraditional risk factors that individually increase the risk of cardiovascular events or total mortality in patients with kidney failure with replacement therapy (KFRT), for example, inflammation and oxidative stress [3,4].

Inflammation, a common complication in MHD patients, is closely related to the occurrence and prognosis of CVD [5]. Several traditional inflammatory cytokines, such as C-reactive protein (CRP), interleukin-6 (IL-6), and tumor necrosis factor-α (TNF-α), have been reported to aggravate kidney function impairment and increase the risk of death in patients with chronic kidney disease (CKD) and MHD [6,7]. However, these traditional inflammatory markers are inconvenient or costly to monitor and are difficult to detect routinely in MHD patients.

The platelet-to-lymphocyte ratio (PLR) is readily available clinical data derived from the complete blood count by dividing the absolute count of platelets by the absolute count of lymphocytes. In recent years, PLR has been used as a new marker of inflammation and has been proven to be independently associated with other inflammatory markers [8] as well as poor prognosis of cardiovascular and tumor diseases [9,10]. PLR has also been independently associated with various kidney-related diseases, such as CKD and acute kidney injury [11,12].

In recent years, studies have reported that PLR is closely related to the prognosis of MHD patients, but the existing studies are limited, and the results are inconsistent [13–15]. The aim of this study was to further evaluate whether PLR is independently associated with all-cause mortality and cardiovascular mortality in patients with MHD and to explore the underlying mechanisms of this relationship with the hope of providing a more concise and cost-effective method for predicting the risk of mortality in patients with MHD.

## 2. Materials and methods

### 2.1. Study design and study population

Patients undergoing regular hemodialysis (HD) in the Blood Purification Center of the General Hospital of Northern Theater Command from January 1, 2014, to December 31, 2014, were retrospectively selected. Inclusion criteria: regular HD treatment ≥3 months; dialysis was performed 3 times/week, 4 h/time; and the dialysis data of the patients were complete. Exclusion criteria: recent infection or use of ­antibiotics; patients with malignant tumors, liver cirrhosis, ­unexplained thrombocytopenia, hematological diseases, autoimmune diseases; a history of steroid or immunosuppressive drugs in the past month; history of surgery or trauma in the past month; active bleeding; patients who underwent kidney transplant or whose status was changed to receiving peritoneal dialysis or who were transferred to another hospital dialysis center.

A total of 303 MHD patients were enrolled according to the inclusion and exclusion criteria. All patients were treated with bicarbonate dialysate and an F80 (Fresenius, Germany) polysulfone membrane dialyzer. Blood flow was 200–350 mL/min, dialysate flow was 500 mL/min, and dialysis was performed three times a week for four hours each time. The ultrafiltration volume of each dialysis corresponded to the target dry body weight that was estimated clinically. The endpoint of follow-up was death or December 31, 2021. The primary endpoints were all-cause death and cardiovascular death. All-cause death was defined as death from any cause. Cardiovascular death included death due to myocardial infarction, heart failure, cardiac arrest, cerebrovascular accident, and peripheral vascular disease [16]. All procedures performed in studies involving human participants were in accordance with the ethical standards of the institutional and/or national research committee and with the 1964 Helsinki Declaration and its later amendments or comparable ethical standards. The study was approved by the Ethics Committee of General Hospital of Northern Theater Command (approval number: Y2022-146).

### 2.2. General information and hematological examination

Demographic data, including age, sex, dialysis duration, body mass index (BMI), primary disease, history of hypertension, diabetes, coronary heart disease, smoking, and use of active vitamin D or phosphate binders, were collected. In the early morning after an overnight fast and before the start of HD, a blood sample was collected from each patient, which was analyzed for white blood cells (WBC), hemoglobin (HGB), lymphocytes, neutrophils, and platelets, neutrophil-to-lymphocyte ratio (NLR), PLR, CRP, serum calcium (Ca), serum phosphorus (P), 25-hydroxyitamin D (25(OH)D), uric acid (UA), urea nitrogen (BUN), creatinine (Cr), albumin (Alb), serum iron (Fe), total iron binding capacity (TIBC), ferritin, β2-microglobulin (β2-MG), and parathyroid hormone (PTH). Blood chemistry parameters were assayed by standardized and automated techniques in the same laboratory. NLR and PLR were calculated from neutrophil, lymphocyte, and platelet counts that were obtained from the baseline hemogram. NLR was defined as the ratio of neutrophil count to lymphocyte count, and PLR was defined as the ratio of platelet count to lymphocyte count.

### 2.3. Statistical analysis

Continuous variables consistent with a normal distribution are expressed as the mean ± standard deviation, and comparisons between groups were performed by independent sample *t*-tests. Continuous variables showed a nonnormal distribution and were represented by the median (lower and upper quartiles), and comparisons between groups were performed by the Mann–Whitney *U* test. Categorical variables are expressed as frequencies or percentages, and the chi-square test was used for comparisons between groups. The receiver operating characteristic (ROC) curve was used to detect the predictive ability of PLR and NLR and the optimal critical values of PLR and NLR were determined to be 107.57 and 2.58, respectively. Kaplan–Meier survival analysis was used to generate survival curves, and the log-rank test was performed to test the differences between survival curves to compare the prognostic ability of PLR and NLR in MHD patients. Univariate and multivariate Cox proportional hazards regression models were used to analyze the risk factors for all-cause mortality and cardiovascular mortality in MHD patients. The prognostic value of PLR and NLR on all-cause mortality and cardiovascular mortality in MHD patients was analyzed by calculating hazard ratios (HRs) and 95% confidence intervals (CIs). The multivariate Cox proportional hazards regression model included variables that were identified as significantly associated with all-cause and cardiovascular mortality in univariate analysis (*p* < .05). IBM SPSS 22.0 was used for statistical analysis. A value of *p* < .05 was considered statistically significant.

## 3. Results

### 3.1. Baseline patient characteristics

**Figure 1.**
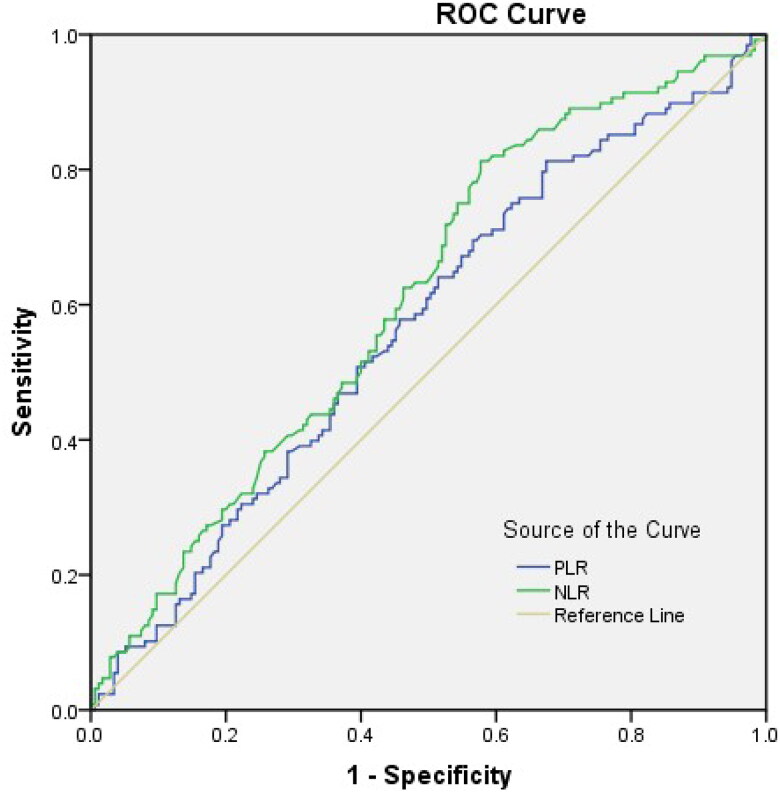
ROC analysis of the relationship between PLR, NLR, and all-cause mortality in MHD patients.

A total of 303 MHD patients were enrolled in this study, with an average age of 53.65 ± 13.17 years, including 184 males (60.73%). The median dialysis duration was 2.0 (1.0, 5.0) years. Various primary diseases were represented, including hypertension (59 cases), diabetes (64 cases), nephritis (157 cases) and others (23 cases). By the end of follow-up, 128 (42.24%) MHD patients had progressed to all-cause death, and 73 (24.09%) MHD patients had progressed to cardiovascular death. To obtain the optimal cutoff value of PLR, we performed ROC curve analysis using the endpoint event as the status variable. As shown in Figure 1, the optimal cutoff value was 107.57 for PLR. Patients were divided into two groups based on this optimal PLR cutoff value. There were 215 patients in the PLR ≥ 107.57 group and 88 patients in the PLR < 107.57 group. Patients in the high-PLR group had lower Fe (*p* = .021), lower β2-MG (*p* = .016), lower lymphocytes (*p* < .001), higher neutrophils (*p* = .049), higher platelets (*p* < .001), and higher NLR (*p* < .001) (Table 1).

**Table 1.** Demographic characteristics and laboratory Measurements.

	Total (*n* = 303)	PLR < 107.57 (*n* = 88)	PLR ≥ 107.57 (*n* = 215)	*p* value
Age, years	53.65 ± 13.17	52.81 ± 13.03	53.99 ± 13.25	.479
Sex, male, n (%)	184 (60.73%)	51 (57.95%)	133 (61.86%)	.527
Dialysis duration, years	2.0 (1.0,5.0)	3.0 (1.0,6.0)	2.0 (1.0,5.0)	.207
Hypertension, n (%)	252 (83.17%)	68 (77.27%)	184 (85.58%)	.079
Diabetes, n (%)	80 (26.4%)	20 (22.73%)	60 (27.91%)	.353
Coronary heart disease, n (%)	128 (42.24%)	41 (46.59%)	87 (40.47%)	.327
Smoking status, n (%)	43 (14.19%)	10 (11.36%)	33 (15.35%)	.367
Using active vitamin D, n (%)	231 (76.24%)	72 (81.82%)	159 (73.95%)	.07
Using phosphorous binders, *n* (%)	256 (84.49%)	77 (87.5%)	179 (83.26%)	.25
WBC, ×10^9^/L	6.3 (5.2,7.7)	6.0 (5.03,7.68)	6.3 (5.3,7.7)	.388
HGB, g/L	106 (96,116)	107.5 (99,116.75)	104 (95,115)	.154
Ca, mmol/L	2.21 (2.11,2.39)	2.2 (2.1,2.38)	2.23 (2.11,2.41)	.476
P, mmol/L	1.96 (1.63,2.4)	1.96 (1.52,2.6)	1.96 (1.64,2.34)	.875
BMI, kg/m^2^	22.68 (20.55,25.12)	22.63 (20.58,25.54)	22.68 (20.52,24.8)	.744
25 (OH)D, ng/ml	18.74 (11.9,24.11)	18.44 (12.66,23.99)	18.89 (11.33,24.47)	.917
UA, mmol/L	458 (401,528)	454.5 (386.75,535.75)	458 (405,519)	.663
BUN, mmol/L	27.1 (22.6,31.6)	27.1 (21.73,31.1)	27.1 (23.4,31.6)	.397
Cr, µmol/L	1002 (835,1186)	1020.5 (831.75,1238.75)	988 (839,1169)	.494
Alb, g/L	40 (37.7,42.3)	40.2 (38.63,42.1)	39.8 (37.6,42.3)	.238
Fe, µmol/L	11.13 (8.39,13.94)	11.95 (9.02,14.23)	10.8 (8.06,13.64)	.021
TIBC, µmol/L	43 (39,49)	43 (39,47.75)	44 (39,50)	.287
Ferritin, ng/ml	182 (70,454)	219 (81.5,565.75)	175 (67,388)	.195
β2-MG, mg/L	29.5 (24.88,35.53)	32.22 (26.35,37.61)	28.55 (24.28,34.35)	.016
PTH, pg/ml	233 (93,485)	256.5 (101,511)	222 (92,458)	.689
CRP, mg/L	3.1 (1.53,5.3)	2.99 (1.35,5.49)	3.1 (1.53,5.21)	.745
Lymphocyte, ×10^9^/L	1.3 (1.0,1.6)	1.5 (1.3,1.9)	1.2 (0.9,1.4)	<.001
Neutrophil, ×10^9^/L	4.1 (3.0,5.2)	3.85 (2.9,4.7)	4.1 (3.1,5.3)	.049
Platelet, ×10^9^/L	172 (141,214)	150 (119,173.5)	186 (152,225)	<.001
NLR	3.22 (2.41,4.31)	2.44 (1.87,3.2)	3.57 (2.69,4.75)	<.001

WBC: leukocyte; HGB: hemoglobin, Ca: serum calcium; P: serum phosphorus; BMI: body mass index; 25 (OH)D: 25 hydroxyvitamin D; UA: uric acid; BUN: urea nitrogen; Cr: creatinine; Alb: albumin; Fe: serum iron; TIBC: total iron binding capacity; β2-MG: β2-microglobulin; PTH: pyrin; CRP: C-reactive protein; NLR: neutrophil-to-lymphocyte ratio; PLR: platelet-to-lymphocyte ratio.

### 3.2. Correlations of PLR and NLR with the prognosis of MHD patients

**Figure 2.**
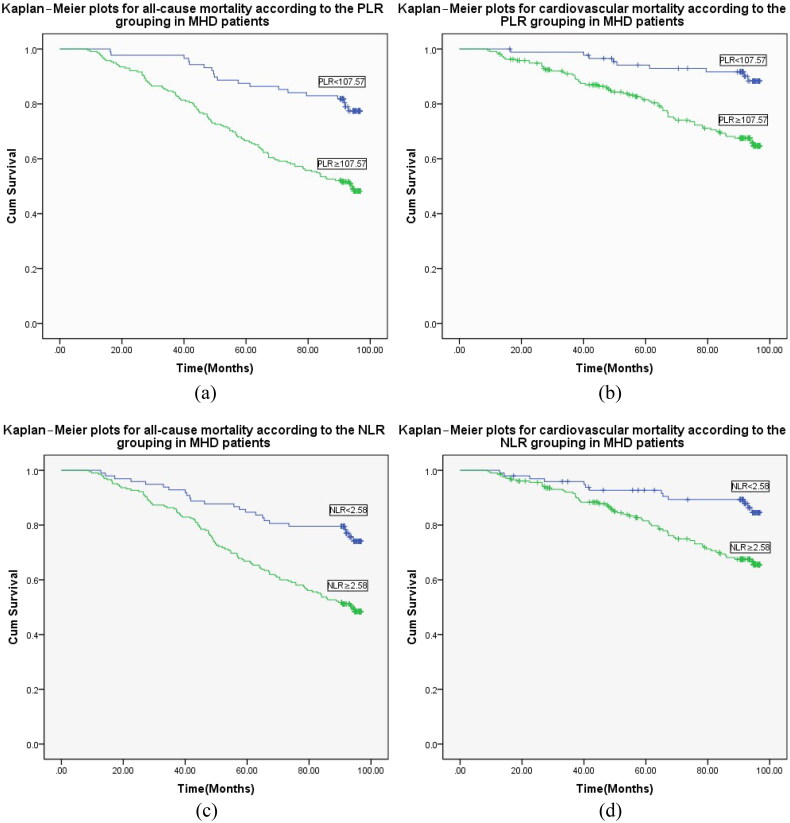
Kaplan–meier plots for all-cause and cardiovascular mortality according to the MHD patient groups. (a) Kaplan–meier plots for all-cause mortality according to PLR grouping in MHD patients (log-rank test, *p* < 0.001). (b) Kaplan–meier plots for cardiovascular mortality according to the PLR grouping in MHD patients (log-rank test, *p* < 0.001). (c) Kaplan–meier plots for all-cause mortality according to the NLR grouping in MHD patients (log-rank test, *p* < 0.001). (d) Kaplan–meier plots for cardiovascular mortality according to the NLR grouping in MHD patients (log-rank test, *p* = 0.001).

Survival analysis revealed that all-cause mortality and cardiovascular mortality in the high-PLR group were significantly higher than those in the low-PLR group (χ2 = 20.860, *p* < .001 and *χ*^2^ = 15.402, *p* < .001). Similarly, the patients were assigned to high-NLR (*n* = 205) and low-NLR (*n* = 98) groups, with the NLR diagnostic threshold of 2.58 as the dividing line. Survival analysis showed significantly higher all-cause mortality and cardiovascular mortality in patients with a high NLR than in those with a low NLR (*χ*^2^ = 17.127, *p* < .001 and *χ*^2^ = 10.964, *p* = .001) (Figure 2).

### 3.3. Multivariable Cox regression analysis was used to determine the independent effects of PLR in predicting all-cause and cardiovascular mortality

Univariate Cox regression analysis revealed that all-cause mortality was associated with age (*p* < .001), diabetes mellitus (*p* < .001), coronary heart disease (*p* = .004), HGB (*p* = .032), albumin (*p* < .001), ferritin (*p* = .02), PLR (*p* < .001), and NLR (*p* < .001) (Table 2). Following adjusted Cox regression analysis, PLR (HR = 2.608, 95% CI 1.579–4.306, *p* < .001) and NLR (HR = 1.634, 95% CI 1.023–2.610, *p* = .04) were still associated with all-cause mortality (Table 2). Univariate and multivariate Cox regression analyses revealed that diabetes (HR = 2.669, 95% CI 1.633–4.363, *p* < .001), coronary heart disease (HR = 1.638, 95% CI 1.013–2.648, *p* = .044), and PLR (HR = 3.379, 95% CI 1.646–6.936, *p* = .001) were factors significantly associated with cardiovascular mortality. We did not find an independent association between the NLR and cardiovascular mortality (Table 3).

**Table 2.** Univariate and multivariate Cox regression analyses of all-cause mortality in MHD patients(*n* = 303).

	Univariate			Multivariate		
	HR	95% CI	*p* value	HR	95% CI	*p* value
Age, years	1.047	1.033–1.062	<.001	1.037	1.021–1.053	<.001
Dialysis duration, years	0.945	0.889–1.003	.064			
Diabetes	3.580	2.519–5.088	<.001	2.457	1.697–3.558	<.001
Coronary heart disease	1.673	1.182–2.367	.004	1.307	0.913–1.869	.144
WBC, ×10^9^/L	0.996	0.973–1.021	.766			
HGB, g/L	0.988	0.976–0.999	.032	0.985	0.973–0.997	.014
Alb, g/L	0.902	0.858–0.948	<.001	0.986	0.933–1.042	.608
CRP, mg/L	1.035	0.996–1.077	.079			
Ferritin, ng/ml	1	1.000–1.001	.02	1	1.000–1.001	.007
PLR ≥ 107.57	2.952	1.813–4.808	<.001	2.608	1.579–4.306	<.001
NLR ≥ 2.58	2.475	1.587–3.860	<.001	1.634	1.023–2.610	.04

Adjusted for age, diabetes, coronary heart disease, and HGB, Alb, and ferritin levels. NLR: neutrophil-to-lymphocyte ratio; PLR: platelet-to-lymphocyte ratio; HR: hazard ratio; CI: confidence intervals; WBC: leukocyte; HGB: hemoglobin; Alb: albumin; CRP: C-reactive protein.

**Table 3.** Univariate and multivariate Cox regression analyses of cardiovascular mortality in MHD patients(*n* = 303).

	Univariate			Multivariate		
	HR	95% CI	*p* Value	HR	95% CI	*p* value
Age, years	1.034	1.016–1.053	<.001	1.019	0.999–1.039	.067
Dialysis duration, years	0.939	0.866–1.018	.126			
Diabetes	3.807	2.391–6.061	<.001	2.669	1.633–4.363	<.001
Coronary heart disease	1.956	1.231–3.106	.004	1.638	1.013–2.648	.044
WBC, ×10^9^/L	1	0.975–1.026	.987			
HGB, g/L	0.994	0.979–1.009	.419			
Alb, g/L	0.911	0.852–0.974	.006	0.973	0.909–1.041	.423
CRP, mg/L	1.045	0.996–1.098	.073			
Ferritin, ng/ml	1	1.000–1.001	.01	1.001	1.000–1.001	.002
PLR ≥ 107.57	3.684	1.832–7.407	<.001	3.379	1.646–6.936	.001
NLR ≥ 2.58	2.650	1.454–4.831	.001	1.606	0.854–3.023	.142

Adjusted for age, diabetes, coronary heart disease, Alb, and ferritin levels. NLR: neutrophil-to-lymphocyte ratio; PLR: platelet-to-lymphocyte ratio; HR: hazard ratio; CI: confidence interval; WBC: leukocyte; HGB: hemoglobin; Alb: albumin; CRP: C-reactive protein.

## 4. Discussion

Inflammation is an important pathogenic mechanism promoting the occurrence and development of CKD and is associated with increased all-cause mortality in CKD patients [17]. Systemic persistent inflammation is also the main cause of uremica-related complications, such as uremica combined with cardiovascular disease, protein and energy consumption, depression, and osteoporosis [5]. Inflammation is also a strong predictor of poor prognosis in HD patients and increases cardiovascular risk and mortality in MHD patients [18].

PLR is used as an informational marker to reflect changes in platelet and lymphocyte counts in acute inflammatory and prethrombotic states [19]. PLR is also considered a marker of systemic inflammation when patients have no obvious infection [20]. NLR, as a novel inflammatory marker, has also become a predictor of poor prognosis in cancer and cardiovascular diseases in the general population [10,21]. Turkey K et al. showed that in KFRT patients, PLR can better predict the inflammatory state of the body than NLR [8]. Given that both thrombocytosis and lymphocytopenia are associated with the degree of systemic inflammation, an index that combines these two factors, PLR, has been proposed as a novel and useful inflammatory marker in clinical practice [22] and is inexpensive and easy to obtain. PLR was originally considered a prognostic factor for cancer [23]. Later, a high PLR was found to be associated with a relatively high inflammatory state and poor prognosis in a variety of diseases, such as CVD [9], hepatocirrhosis [24], rheumatic diseases [19], and CKD [11].

At present, there are few studies on the relationship between PLR, NLR, and all-cause and cardiovascular mortality in MHD patients, and the results are still controversial. There is no clear cutoff value standard for PLR and NLR in the general population or dialysis population. Previous studies have different cutoff values for predicting all-cause mortality and cardiovascular mortality in MHD patients with PLR and NLR due to differences in race, inclusion and exclusion criteria, or cutoff value methods. For example, previous studies have suggested that PLR ≥ 130.4 and PLR > 142.38 are predictive factors for all-cause mortality and cardiovascular mortality in MHD patients, respectively. However, in these experiments, the median PLR was used as the cutoff value [13,14]. In a study on continuous ambulatory peritoneal dialysis patients, PLR ≥ 118.53 was a related predictor of cardiovascular disease. The receiver operating characteristic was used to select the cutoff value in the experiment [25]. Similarly, there are similar issues with the value of NLR [14,26]. Therefore, more experimental studies are needed to determine the truncation values of PLR and NLR.

A prospective cohort study of 80 MHD patients conducted by Yaprak M et al. found that although NLR and PLR were associated with all-cause mortality in prevalent HD patients, only PLR independently predicted all-cause mortality in these populations [13]. Zhang et al. conducted a 71-month follow-up of 360 MHD patients and found that higher NLR levels were independently associated with all-cause mortality, while higher PLR levels were potentially predictive of cardiovascular mortality [14]. A retrospective study conducted by Elizabeth Russu et al. established that the NLR, MLR, and PLR determined at hospital admission had a strong predictive capacity for all-cause 30-day mortality in kidney failure patients who required kidney replacement therapy for at least 6 months. Elevated values of the ratios were also associated with longer hospital stays and more dialysis sessions per patient [27]. After a retrospective analysis of 108,548 MHD patients, Catabay C et al. found that the NLR was increasingly associated with high mortality, but the PLR was weakly associated with mortality and had little benefit in its prediction. However, the study’s data came from administrative databases at large dialysis facilities and did not rule out conditions such as infection, malnutrition, central catheters, stress, metabolic syndrome, and atherosclerosis that affect PLR and NLR levels. Moreover, the study included only incident HD patients, and it is unclear whether the findings are generalizable to all MHD patients or peritoneal dialysis patients [15]. Han Li et al. conducted a study on the relationship between the NLR, cardiovascular risk indicators, and mortality in 268 HD patients. After adjusting for other risk factors, a high NLR is an independent predictor of all-cause mortality and cardiovascular mortality in HD patients. However, this study did not compare the NLR with other simple inflammatory markers, such as WBC count and PLR. Therefore, this study cannot prove the best biomarker for predicting cardiovascular risk factors and mortality in HD patients, nor can it rule out the impact of PLR on the final outcome [26]. In this study, PLR and NLR were risk factors for all-cause mortality in MHD patients, and the results were still significantly correlated after adjusting for confounding factors. PLR was also found to be an independent risk factor for cardiovascular mortality in MHD patients, but NLR was not. Our study showed that PLR was associated with both all-cause and cardiovascular mortality in MHD patients. However, in our study, no association was found between NLR and cardiovascular mortality in MHD patients. The effect of platelet activation on the poor prognosis of cardiovascular disease may explain this conclusion [28].

Although the exact mechanism by which high PLR may contribute to high all-cause and cardiovascular mortality in MHD patients is unclear, inflammation may play an important role. As a new inflammatory marker, PLR can reflect the inflammatory state of many diseases [9,19,24]. Microinflammation is an important factor in the pathogenesis of CVD in HD patients. The microinflammatory state causes vascular endothelial dysfunction and increases the expression of vascular endothelial adhesion molecule (VCAM-1), which is related to increased volume load and extracellular fluid overloading in HD patients [29]. On the other hand, in a study of the relationship between platelets and atherosclerosis, it was found that the high shear force of blood flow could induce the activation of platelets and enhance the adhesion of platelets to endothelial cells [30]. After platelet activation, a large amount of stromal cell-derived factor-1 can be expressed, which may induce CD34+ stem cells to differentiate into macrophages and foam cells, leading to leukocyte chemotaxis toward vascular endothelial cells in concert with monocyte exudation and formation of foam cells under the vascular intima, thereby causing atherosclerosis [31]. Because of the important role leukocytes play in the development of atherosclerosis and its complications [32], leukocytes, as a typical inflammatory marker, are associated with several coronary risk factors [33]. Previous studies have shown that PLR is superior to white blood cell count in predicting adverse outcomes in a variety of cardiovascular diseases [34]. In addition, as an inflammatory marker, CRP has been associated with a higher risk of death in dialysis patients, but some studies have shown that the relationship between dialysis outcomes and CRP is not significant. The results of current studies on the prognostic value of CRP in dialysis patients are inconsistent [35]. There is currently no comparative study on PLR or NLR and CRP in predicting mortality in dialysis patients. However, studies have shown that the NLR is superior to CRP in predicting the occurrence of liver fibrosis and differentiated thyroid cancer [36,37]. Our study did not find an association between WBC, CRP, and all-cause and cardiovascular mortality in MHD patients, which may be because we excluded patients with definite infection. More research is needed to further clarify which PLR, NLR, and CRP are more advantageous in evaluating the prognosis of dialysis patients.

Platelets, as a type of inflammatory nuclear cell, play a beneficial role in the development of atherosclerosis [38]. During the inflammatory process in which platelets react with vascular endothelial cells, various inflammatory mediators, such as IL-1, IL-6, and TNF-α, are released to stimulate macrophage proliferation and further increase circulating platelet levels [39,40]. Activated platelets enhance the above inflammatory process by secreting cytokines and chemokines and further promote the development of atherosclerosis. Atherosclerosis is a major cause of CVD. However, CVD is one of the leading causes of death in MHD patients [41,42]. In addition to participating in the inflammatory process of atherosclerosis, platelets play an important role in atherosclerotic plaque rupture and subsequent local thrombosis. After atherosclerotic plaque rupture, extracellular matrix proteins in the plaque are exposed, and circulating platelets accumulate rapidly at the exposed site, leading to local thrombosis. At the same time, activated platelets secrete matrix metalloproteinases (MMPs), which promote extracellular matrix degradation and local activation of factors conducive to plaque rupture and thrombosis [43,44], resulting in myocardial infarction or stroke. High platelet counts are usually associated with enhanced platelet activity [8]. Studies have shown that in the absence of absolute thrombocytosis, a higher PLR is associated with thrombosis and increased inflammation, possibly caused by enhanced platelet activity [45].

Finally, importantly, a higher PLR may not be due to increased platelet activity or a relative increase in the number of platelets but also a relative decrease in the number of lymphocytes. A previous study suggested that low lymphocyte levels represented a suppression of the immune response and are associated with poor outcomes [46].

In dialysis patients, advanced age, diabetes, and other traditional risk factors cannot explain the high mortality rate [2]. Diabetes also increases the main factors that lead to an increase in cardiovascular and noncardiovascular mortality, including inflammatory stress and oxidative stress in dialysis patients [47]. Serum ferritin is an acute phase reactant and a marker of acute and chronic inflammation. It exhibits nonspecific elevation under various inflammatory conditions, including chronic kidney disease, acute infection, and malignant tumors [48]. High serum ferritin levels are associated with an increased risk of all-cause death in MHD patients [49]. If the level of serum ferritin in HD patients increases, the possibility of potential inflammation and iron overload should be considered [50]. After excluding the influence of age, traditional diabetes factors, and ferritin indicators on the prognosis of MHD patients, our study still found that PLR and NLR played a role in the prognosis of death in MHD patients.

Currently, in clinical practice, some representative inflammatory markers, including IL-6 and TNF-α, are difficult to routinely detect in MHD patients, are inconvenient and expensive, and cannot be carried out routinely in every hospital and applied to every MHD patient. Our research findings suggest that PLR, as a simple, easily obtainable, and cost-effective new inflammatory marker, can be used by clinical doctors to assess the micro inflammatory state and prognosis evaluation related to cardiovascular and all-cause mortality in MHD patients when other expensive inflammatory marker tests are not available.

Our study has some limitations. First, this was a single-center retrospective study with a limited number of samples. Second, the study examined the values of hematological indicators in MHD patients at the time of enrollment only without dynamically monitoring their changes. Third, we did not validate our findings in other dialysis centers. Finally, we did not compare the value of the NLR or PLR with that of other inflammatory markers, such as IL-6 and TNF-α, which may weaken the conclusions about the prognostic value of the NLR and PLR.

## 5. Conclusions

Inflammation is a strong predictor of poor prognosis in MHD patients. As a new inflammatory marker in recent years, PLR is an independent predictor of all-cause mortality and cardiovascular mortality in MHD patients.

## Data Availability

The datasets used in this study are available from the corresponding author on reasonable request.
